# Optimizing Finishing Pig Performance and Sustainability: The Role of Protein Levels and Eco-Friendly Additive

**DOI:** 10.3390/ani15091248

**Published:** 2025-04-28

**Authors:** Weihan Zhao, Kyejin Lee, Inho Kim

**Affiliations:** 1 Department of Animal Biotechnology, Dankook University, Cheonan 31187, Republic of Korea; zhaoweihan57@gmail.com (W.Z.); jookyjin1234@naver.com (K.L.); 2 Smart Animal Bio Institute, Dankook University, Cheonan 31116, Republic of Korea

**Keywords:** eco-friendly additive, meat quality, pig nutrition, low-protein diet, finishing pig

## Abstract

This study contributes to promoting green farming and achieving sustainable pork production. Especially under the conditions of resource scarcity and rising environmental demands, efficient and eco-friendly feeding strategies have become key to industry development. We evaluated the effects of supplementing an eco-friendly additive (EFA) in diets with normal and low protein (−2% CP) levels on growth performance, nutrient digestibility, gas emission, fecal score, meat quality, and blood profile in finishing pigs. In this 10-week (70-day and 7-day adaptation period) feeding experiment, 200 crossbred pigs [Duroc × (Landrace × Yorkshire)] with an initial average body weight (BW) of 55.05 ± 3.35 kg were used. The pigs were randomly assigned to four treatment groups in a 2 × 2 factorial arrangement, with five pigs per replicate group, including two gilts and three barrows per pen, and each treatment group was repeated 10 times. The experimental treatments included the following: two protein levels (normal CP and −2% CP) and two EFA levels (0% and 0.5% EFA). The results showed that pigs fed high-protein + EFA diets had a significantly higher ADG from weeks 0–5 compared to the high-protein control group (*p* < 0.05). From weeks 5–10 and overall, both high-protein + EFA and low-protein + EFA groups had a higher ADG than the low-protein − EFA group (*p* < 0.05). At week 10, the low-protein + EFA group showed significantly higher nitrogen digestibility and significantly lower ammonia emissions compared to the high-protein − EFA group (*p* < 0.05). Both EFA supplementation and protein level had significant effects on ammonia emissions. The fecal score was not significantly affected (*p* > 0.05). In blood profiles, NPY was higher in the high-protein + EFA group than in the low-protein − EFA group (*p* < 0.05). In terms of meat quality, both EFA-supplemented groups had a higher WHC compared to the low-protein − EFA group, while the high-protein + EFA group had a greater LMA (*p* < 0.05) and lower drip loss on day 7 after slaughter (*p* < 0.05). In conclusion, supplementing low-protein diets with EFA can effectively enhance the growth performance of finishing pigs, mitigate environmental pollution, and offer feeding advantages while lowering feed costs.

## Simple Summary

In modern pig farming, it is vital to improve growth performance while reducing environmental impacts and feed costs. However, production systems often face challenges with nutrient utilization and waste management. For this reason, researchers are exploring alternative feed additives to improve efficiency and sustainability. Several studies have shown that eco-friendly additives can improve growth and digestion, but their effects at different dietary protein levels remain unclear. Therefore, this experiment evaluated the effects of adding eco-friendly additives to normal and low-protein diets on finishing pigs. Our results confirmed that eco-friendly additives were effective in improving growth performance, nutrient digestibility, and meat quality while reducing ammonia emissions, making it a beneficial and sustainable feed additive.

## 1. Introduction

The use of antibiotics in pig farming has a history of over 50 years, but due to issues of resistance and residues, many countries have restricted or banned their use [1]. Against this background, although the use of antibiotics in traditional livestock farming has significantly decreased in recent years, the level of antibiotic use in pig production remains relatively high for the purposes of promoting animal growth and reducing disease incidence [2]. As a result, nutritionists have been looking for some alternatives to antibiotics [3]. For instance, the EFA used in this study is a blend of glyconutrients, β-glucans, various enzymes, and a complex mix of probiotics. It serves as an environmentally friendly antibiotic alternative and a fermented feed additive designed to enhance livestock immune function and digestibility, thereby preventing diseases and improving production efficiency. Glyconutrients, bioactive carbohydrates primarily derived from plant-based sugars, including monosaccharides, oligosaccharides, and polysaccharides exhibit strong anti-inflammatory and antimicrobial properties, support energy metabolism and overall health in monogastric animals, and, when properly added to feed, have been shown to enhance livestock growth and development [4]. Furthermore, previous studies have demonstrated that dietary glyconutrient inclusion improves growth performance in growing pigs and enhances pork quality [5]. As structural glucose polymers, β-glucans are naturally found in the cell walls of fungi, yeast, bacteria, algae, seaweed, and cereals. [6]. Previous research indicates that supplementing pig diets with 50 mg/kg of β-glucans can promote gut health and strengthen immune function [7]. The enzymes produced during fermentation, such as proteases, cellulases, and amylases, play a crucial role in breaking down proteins, fats, and starches, thereby enhancing nutrient digestibility and improving feed efficiency [8]. In young pigs, these enzymes are particularly beneficial, as they help compensate for immature digestive enzyme secretion, reduce the risk of post-weaning diarrhea, and support overall gut health [9]. Probiotics are gaining recognition as an effective alternative to in-feed antibiotics due to their ability to modulate gut microbiota, suppress pathogenic bacteria, reduce diarrhea incidence, boost immunity, and optimize feed efficiency [10]. Recent studies have shown that the addition of probiotic-fermented rice bran to swine diets significantly increases the ADG and reduces the feed conversion ratio (FCR), while also improving gut microbiota diversity, nutrient absorption, intestinal barrier function and reducing inflammatory responses [11]. Beyond individual animal health, pig farming faces growing environmental concerns related to nitrogen compound excretion and pollution while also grappling with the limited availability and high costs of high-quality protein sources [12]. Lowering dietary protein content has been proposed as a strategy to mitigate these issues by reducing the dependence on protein-rich feed ingredients while supporting intestinal health and sustainable production practices [13]. The aim of this study is to evaluate the effects of adding an EFA to diets with normal and low protein levels (−2% CP) on growth performance, nutrient digestibility, gas emission, fecal score, meat quality, and blood profile in finishing pigs.

## 2. Materials and Methods

### 2.1. Ethical Statement

All animal experimental protocols were reviewed and approved by the Animal Care and Use Committee of Dankook University (Protocol Number: DK-2-2422; Approval Date: 14 June 2024).

### 2.2. Experimental Material

The EFA used in this study was obtained from a commercial company (Glycozyme; JM Bio Co., Ltd., Daegu, Republic of Korea). This product comprises four functional composite components: glyconutrients, β-glucans, various enzymes (protease, cellulase, and amylase), and a complex probiotic blend (*Lactobacillus plantarum*, *Bacillus subtilis*, and *Saccharomyces cerevisiae*). Specific nutrients are shown in Table 1.

### 2.3. Experimental Design, Animals, and Housing

**Figure 1:**
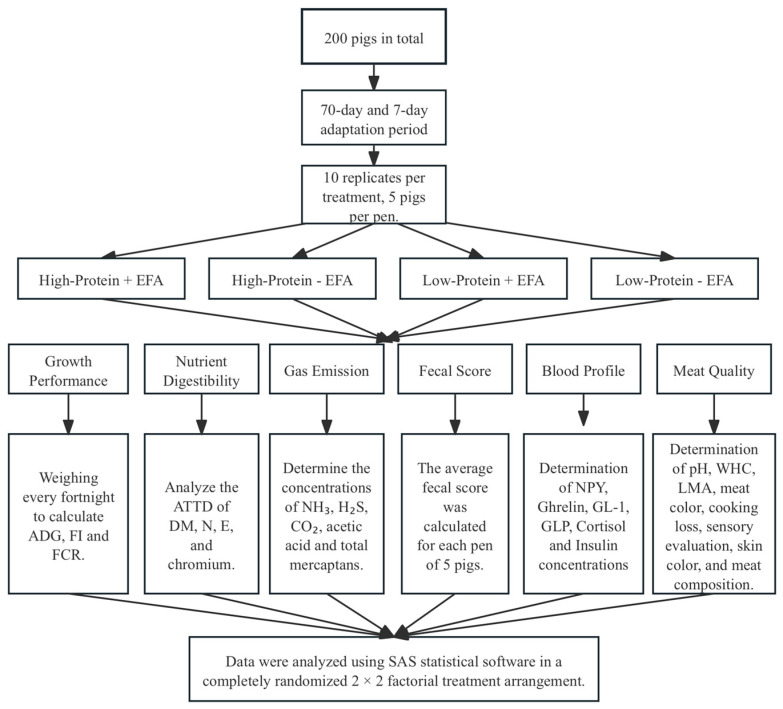
Experimental flow design diagram. Abbreviation: EFA: eco-friendly additive; ADG: average daily gain; ADFI: average daily feed intake; FCR: feed conversion ratio; DM: dry matter; N: nitrogen; E: energy; NH_3_: ammonia; H_2_S: hydrogen sulfide; CO_2_: carbon dioxide; NPY: neuropeptide Y; GLP-1: Glucagon-Like Peptide-1; GIP: gastric inhibitory polypeptide; WHC: water holding-capacity; LMA: *longissimus* muscle area.

In this 10-week (70-day and 7-day adaptation period) feeding experiment, 200 crossbred pigs [Duroc × (Landrace × Yorkshire)] with an initial average BW of 55.05 ± 3.35 kg were grouped by BW and randomly assigned to four treatment groups in a randomized block design. Each replicate group consisted of 5 pigs, with 2 gilts and 3 barrows per pen, and each treatment was repeated 10 times. The experimental treatments included two protein levels (normal CP and −2% CP) and two EFA levels (0% and 0.5% EFA) in a 2 × 2 factorial arrangement. The experimental design is shown in Figure 1. The experimental diets were formulated based on the nutritional requirements specified by the National Research Council (NRC) 2012 [14] to meet or exceed the nutritional needs of finishing pigs (Table 2). The pigs were housed in a temperature-controlled environment with plastic slatted flooring and a mechanical ventilation system. Each pen, measuring 1.8 × 1.8 m, was equipped with an automatic feeding system and nipple drinkers, ensuring free access to feed and water throughout the experiment. Additionally, the facility employed an automated mechanical ventilation system, with 12 h of artificial lighting per day. The room temperature was maintained at 21.5 °C. The facility was monitored three times daily (at 9:00 AM, 2:00 PM, and 7:00 PM) to check for water leakage, ensure adequate feed in the feeders, and address any potential health issues.

### 2.4. Growth Performance

Throughout the 10-week trial, BW was recorded individually at the start and at weeks 5 and 10 to calculate body weight gain (BWG) and ADG by treatment. Simultaneously, FI and refusals were measured to determine average daily feed intake (ADFI) and FCR.

$$FCR=\frac{Feed\;Intake\left(kg\right)}{Weight\;Gain\left(kg\right)}$$

### 2.5. Nutrient Digestibility

To estimate the apparent total tract digestibility (ATTD), 0.20% chromium oxide (Cr_2_O_3_) was added to the diet 7 days prior to fecal collection. At the end of the experiment, fecal samples were randomly collected from two pigs (one male and one female) per pen by rectal massage and pooled by pen. Feed samples were collected and preserved weekly. At week 10, these samples were combined into a composite sample. The feed and fecal samples were then stored at −20 °C until the laboratory analysis for the ATTD of dry matter (DM), nitrogen (N), energy (E), and chromium absorption. Prior to chemical analysis, all feed and fecal samples were dehydrated at 70 °C for 72 h and then finely ground and sieved through a 1 mm sieve to obtain a homogeneous sample. Following the guidelines of the Association of Official Analytical Chemists [15], the nutrient digestibility of DM, N, and E and chromium absorption in feed and fecal samples were analyzed following the method used by Williams et al. [16]. The chromium concentration in the samples was measured using a UV spectrophotometer (Optizen POP, Seoul, Republic of Korea). E was measured by an oxygen bomb calorimeter (Parr 6100 instrument Co., Moline, IL, USA). N was analyzed with a Kjeltec 2300 nitrogen analyzer (Foss Tecator AB, Hillerød, Denmark). The ATTD was estimated using the following formula:

$$ATTD\left(\%\right)=(1-\frac{N_{f}\times C_{d}}{N_{d}\times C_{f}})\times 100$$

where N_f_ indicated concentration in feces (% DM), C_d_ indicated chromium concentration in diets (% DM), N_d_ indicated nutrient concentration in diets (% DM), and C_f_ indicated chromium concentration in feces (% DM).

### 2.6. Gas Emission

At the conclusion of the trial, fresh feces and urine were randomly sampled from at least two pigs (one gilt and one barrow) in each pen. The urine was funneled into a collection bucket positioned beneath the cages. All collected samples were immediately sealed and stored at −4 °C to preserve their integrity until analysis. According to the method outlined by Nguyen et al. [17], fecal and urine samples from each pen were thoroughly mixed separately and then combined to form a fecal–urine slurry. A total of 150 g of feces and 150 g of urine were mixed at a 1:1 ratio on a wet weight basis and stored in 2.6 L plastic boxes, with two replicates per pen. A small hole was created in the middle of one sidewall of each plastic box and sealed with adhesive tape to maintain anaerobic conditions. The samples were allowed to ferment at room temperature (25 °C) for 7 days, and gas concentrations were measured on day 7 of fermentation. Before measurement, the fecal–urine slurry was manually shaken for approximately 30 s to break any crust formation on the surface and ensure sample homogeneity. During measurement, the adhesive tape sealing the plastic box was punctured, and the headspace gas was collected at a rate of 100 mL/min approximately 2.0 cm above the sample surface. The concentrations of ammonia (NH_3_), hydrogen sulfide (H_2_S), carbon dioxide (CO_2_), acetic acid, and total mercaptans were determined using Gastec detector tubes (No. 3La, No. 4LK, and No. 70L; Gastec Corporation, San Diego, CA, USA)

### 2.7. Fecal Score

At the beginning of the experiment and throughout the 10-week trial period, fecal scores were evaluated using a 5-point scoring system according to the method described by Wang et al. [18], with the average score calculated from five pigs per pen. Stool consistency was evaluated using a five-point scoring system: 1 = hard, dry pellets; 2 = firm, well-formed stool; 3 = moist, shaped stool; 4 = unformed, soft stool conforming to the container; and 5 = watery, liquid stool. Scores were assigned per pen based on visual assessment of individual pigs and overall fecal appearance. All pigs were fed a mash-type diet.

### 2.8. Blood Profile

At the beginning and end of the experiment, the blood samples were randomly collected from the cervical vein of 4 pigs from each treatment group into tripotassium ethylenediaminetetraacetate (K_3_EDTA) vacuum tubes and clot activator vacuum tubes (Becton Dickinson Vacutainer Systems, Franklin Lakes, NJ, USA). Serum cortisol was measured using a radioimmunoassay (RIA) kit (Diagnostic Products Co., Los Angeles, NJ, USA). The concentration of Ghrelin was measured according to the instructions of the kit (Jiancheng Bioengineering Institute, Nanjing, China). Neuropeptide Y (NPY) was measured using an NPY ELISA kit (Shanghai Bohu Biological Technology Co., Ltd., Shanghai, China). Insulin was measured using a pig ELISA kit (Mercodia, Uppsala, Sweden). GLP-1 and GIP were measured using Millipore (Billerica, MA, USA) ELISA kits, following the manufacturer’s instructions.

### 2.9. Meat Quality

After 24 h of chilling at 2 °C, a portion of the right loin was obtained by a perpendicular cut between the 10th and 11th ribs. Prior to the meat quality analysis, the samples were thawed at room temperature. The meat and skin color parameters—lightness (L), redness (a), and yellowness (b*)—were assessed using a Minolta CR410 chroma meter (Konica Minolta Sensing, Inc., Osaka, Japan). The sensory traits, including color, marbling, and firmness, were evaluated in accordance with the guidelines established by the National Pork Producers Council [19]. Duplicate pH values were determined for each sample using a pH meter (Fisher Scientific, Pittsburgh, PA, USA). The water-holding capacity (WHC) was assessed following the method described previously [20]. In brief, 0.3 g of meat was compressed under 3000 psi for 3 min on a 125 mm diameter filter paper. The areas of meat and expelled moisture were measured using a digitizing area-line sensor (MT-10S, M.T. Precision Co., Ltd., Tokyo, Japan), and the WHC was expressed as the ratio of moisture area to meat area (a smaller ratio indicates a greater WHC). The LMA was quantified by tracing its cross-sectional surface at the 10th rib using the same sensor. For the calculation of cooking losses, the procedure outlined by Sullivan et al. [21] was carried out by placing 4 g of meat samples in plastic bags and heated in a water bath at 100 °C for 5 min followed by cooling to room temperature. The cooking loss was calculated as:

$$Cooking\;loss=\frac{sample\;weight\;before\;cooking-sample\;weight\;after\;cooking}{sample\;weight\;before\;cooking}\times 100.$$

The drip loss was determined using approximately 4.5 g of meat sample following the plastic bag method. On days 1, 3, 5, and 7, the samples were removed, blotted dry with paper towels, and weighed. The difference in weight between the initial and subsequent measurements was used to calculate the drip loss.

### 2.10. Statistical Analysis

The data were analyzed using SAS 9.4 statistical software (SAS Inst. Inc., Cary, NC, USA, 2002) with a completely randomized 2 × 2 factorial treatment arrangement, where the cage was the experimental unit. The main effects of EFA and protein content, as well as their interaction, were tested. The significance level was set at *p* < 0.05, and 0.05 < *p* < 0.10 was considered a trend.

## 3. Results

The effects of dietary treatments on growth performance are shown in Table 3. No significant differences in BW were observed among the treatment groups (*p* > 0.05). However, pigs in the low-protein + EFA group showed a significantly higher BW at week 10 compared to those in the low-protein − EFA group. Specifically, the body weight of the low-protein + EFA group (116.86 kg) was 0.65% higher than that of the high-protein − EFA group (116.11 kg), indicating that the addition of an EFA can alleviate the negative impact of a low-protein diet on the growth of finishing pigs.

During weeks 0–5, pigs in the high-protein + EFA group had a significantly higher ADG compared to the high-protein control group (*p* < 0.05). From weeks 5 to 10 and over the entire experimental period, both the high-protein + EFA and low-protein + EFA groups showed significantly increased ADG compared to the low-protein − EFA group (*p* < 0.05). There was no significant interaction between the dietary protein level and EFA supplementation on the growth performance of finishing pigs (*p* > 0.05).

**Figure 2:**
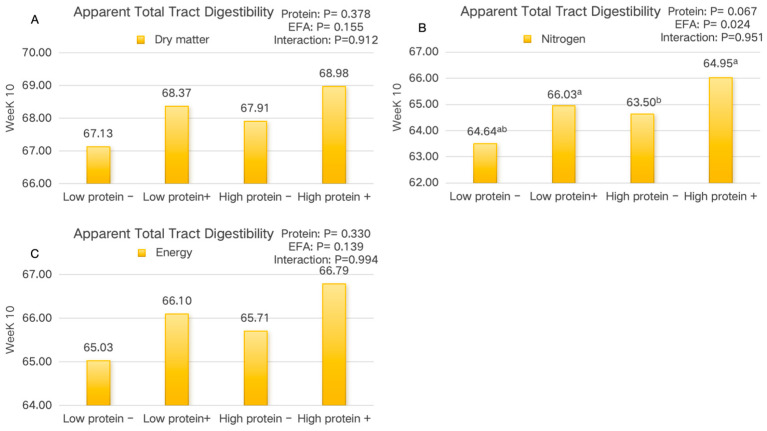
The effect of the addition of EFA to diets with varying protein levels on the apparent total tract digestibility of nutrients in finishing pigs. (**A**) Dry matter digestibility; (**B**) nitrogen digestibility; (**C**) energy digestibility; (−): 0% EFA; (+): add 0.5% EFA; ^a,b^ Means in the same row with different superscripts differ significantly (*p* < 0.05).

The effects of dietary treatments on the ATTD are shown in Figure 2. In week 10, compared with the high-protein − EFA low-protein + EFA group, pigs in the low-protein + EFA group showed a significantly higher nitrogen digestibility (*p* < 0.05). There was no significant interaction between the dietary protein level and EFA supplementation on the ATTD of finishing pigs (*p* > 0.05).

The effects of dietary treatments on gas emissions are shown in Table 4. In week 10, NH₃ emission was significantly lower in the low-protein + EFA group compared to the high-protein − EFA group (*p* < 0.05), indicating that the addition of an EFA helps reduce ammonia emissions. Furthermore, the main effects of EFA addition and protein levels on NH₃ were significant (*p* < 0.05). Additionally, there was no significant interaction between dietary protein levels and the addition of EFA on gas emissions in finishing pigs (*p* > 0.05).

The effects of dietary treatments on fecal scores are shown in Table 5. In this study, dietary protein levels and the addition of an EFA had no significant effects or interactions on fecal scores in finishing pigs (*p* > 0.05).

**Figure 3:**
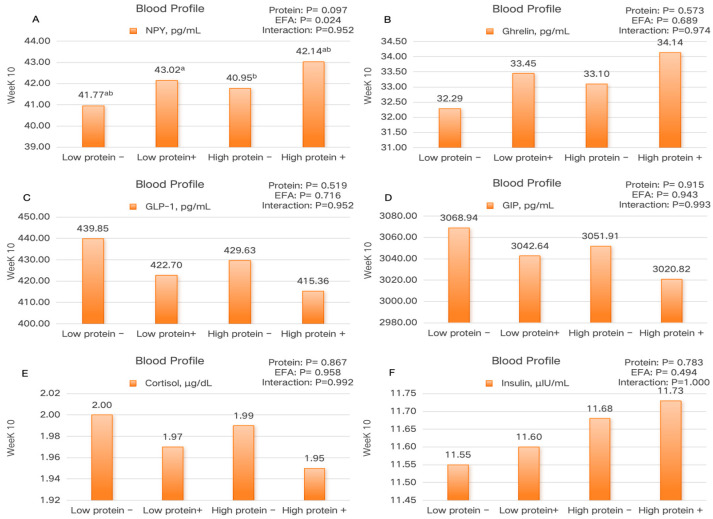
The effect of the addition of EFA to diets with varying protein levels on the blood profile in finishing pigs. (**A**) Neuropeptide Y; (**B**) Ghrelin; (**C**) Glucagon-Like Peptide-1; (**D**) gastric inhibitory polypeptide; (**E**) cortisol; (**F**) insulin; (−): 0% EFA; (+): add 0.5% EFA; ^a,b^ Means in the same row with different superscripts differ significantly (*p* < 0.05).

The effects of dietary treatments on blood profiles are shown in Figure 3. In week 10, regarding the blood profile, the NPY concentration in pigs from the high-protein + EFA group was significantly higher than that in the low-protein − EFA group (*p* < 0.05). Additionally, there was no significant interaction between dietary protein levels and the addition of EFA on gas emissions in finishing pigs (*p* > 0.05).

The effects of dietary treatments on meat quality are shown in Table 6. In terms of meat quality, the WHC of pigs in both the high-protein + EFA group and the low-protein + EFA group was significantly higher compared to the low-protein − EFA group (*p* < 0.05). The LMA was also significantly higher in the high-protein + EFA group than in the low-protein − EFA group (*p* < 0.05). On day 7, drip loss in the low-protein − EFA group was significantly higher compared to the high-protein + EFA group (*p* < 0.05). Additionally, there was no significant interaction between dietary protein levels and the addition of the EFA on gas emissions in finishing pigs (*p* > 0.05).

## 4. Discussion

Studies have shown that the addition of probiotics to the diet can significantly increase the ADG of weaned pigs, with effects comparable to those observed in pigs fed antibiotic-added diets [22]. Additionally, research has indicated that the addition of β-glucan can improve the ADG and ADFI of growing–finishing pigs [23]. Furthermore, previous studies have demonstrated that the addition of a combination of protease, cellulase, and amylase to the diet of growing pigs can significantly enhance growth performance by increasing the ADG and BW [24]. In this study, the addition of an EFA to the growing pig diet significantly improved the ADG during weeks 0–5, weeks 5–10, and the entire experimental period, while ADFI and BW showed no significant statistical changes. This may be because during the later stage of growth, feed intake tends to stabilize, and pigs adjust their intake based on the metabolic demands to maintain energy balance. Although no significant statistical differences were observed in the final body weight among the treatment groups, the trend in body weight gain was consistent with other growth performance indicators. Notably, the final body weight of the low-protein + EFA group was 116.86 kg, even higher than that of the high-protein control group (116.11 kg), showing an increase of about 0.65%. This trend was consistent with the significant increase in the ADG during weeks 5–10 and the entire experimental period. Therefore, the significant improvement in the ADG indirectly reflects the positive role of the EFA in promoting growth, particularly under low-protein dietary conditions, with growth increases of 2.29% (low-protein group) > 1.46% (high-protein group).

It is widely believed that changes in nitrogen digestibility can influence fecal composition and contribute to the emission of harmful gases from livestock production, such as NH_3_, H_2_S, and other gaseous compounds. These emissions not only lead to environmental pollution but may also pose risks to both human and animal health [25]. Some studies have found that the addition of a multi-strain probiotic to the feed can enhance protein and amino acid digestion, lower intestinal pH, improve nitrogen digestibility in finishing pigs, and reduce ammonia emissions [26]. Similarly, some researchers have reported that the addition of a complex enzyme additive to the diet of growing pigs can improve protein and nitrogen digestibility, increase feed and microbial utilization, and consequently reduce ammonia emissions [27]. The results of this study are generally consistent with the aforementioned findings. The observed improvement in nitrogen digestibility in finishing pigs following the addition of an EFA may contribute to a reduction in NH_3_ emissions. This could be due to the enhanced microbial utilization of protein in the gut, which increases nitrogen conversion rates while alleviating the burden on the urea cycle [28]. As a result, the concentration of NH_3_ in the blood may be reduced, potentially leading to lower NH_3_ emissions in the feces [29]. However, other studies have reported that the addition of a combination of probiotics and β-glucan to feed had no significant effect on nitrogen digestibility or ammonia emissions in finishing pigs [5]. This discrepancy may be attributed to differences in gas measurement methods, as well as variations in environmental factors such as temperature and humidity.

High-quality pork must not only meet consumer expectations regarding color, tenderness, and flavor but also relate directly to its nutritional value and food safety. Studies have reported that the addition of a combination of probiotics and glyconutrients to sow diets can reduce the drip loss in offspring carcasses and increase the LMA, thereby improving meat quality [30]. Additionally, the addition of probiotics to feed has been shown to enhance gut microbiota, strengthen immune function, and promote nutrient absorption, ultimately improving the muscle quality and WHC [31]. The findings of this study are largely consistent with these reports, as the EFA addition group exhibited significantly increased WHC and LMA, along with a significant reduction in drip loss. However, other studies have suggested that the addition of EFA has no significant effect on the WHC, LMA, or drip loss in growing pigs [32]. This discrepancy may be attributed to the fact that growing pigs are in a rapid developmental phase with different nutritional requirements compared to finishing pigs. At this stage, β-glucans, glyconutrients, enzyme preparations, and probiotics may have a less pronounced impact on meat quality than the finishing phase.

As an important neurotransmitter, NPY plays a crucial role in regulating appetite, energy metabolism, fat deposition, and growth performance. In this study, the addition of an EFA significantly increased NPY levels, prompting further investigation into its role in the growth and development of finishing pigs. Previous research has shown that elevated NPY levels can stimulate appetite and feed intake, thereby enhancing animal growth performance [33]. This finding aligns with our study, where the addition of enzymes and probiotics improved gut microbiota, enhanced nutrient absorption and utilization efficiency, and ultimately promoted growth rate. Furthermore, after the NPY levels increased, we observed a significant improvement in pork water-holding capacity and a reduction in drip loss, suggesting that NPY may contribute to improved meat quality by enhancing water retention and tenderness. NPY is recognized as a potent orexigenic factor that stimulates feeding behavior in non-mammalian species in a dose-dependent manner [34]. However, in this study, although NPY levels increased, the ADFI did not show a significant change. This may be attributed to the dual role of NPY in regulating both appetite and energy metabolism. Some studies suggest that when an animal’s energy intake and metabolic demands are balanced, the increase in NPY levels may not significantly elevate feed intake due to the regulatory effects of energy metabolism’s negative feedback mechanisms, where elevated NPY can be offset by other metabolic signals, thus maintaining overall feed intake stability [35,36]. Additionally, if the feed quality or palatability is suboptimal, the increased NPY levels may enhance appetite but not necessarily lead to greater feed consumption, which could explain the unchanged ADFI observed in this study.

In this study, the low-protein diet significantly reduced ammonia emissions in finishing pigs, primarily due to the regulatory mechanisms of nitrogen metabolism. The main source of ammonia is the unused nitrogen excreted in feces and urine, particularly from the breakdown of urea. Research has shown that low-protein diets reduce the total nitrogen supply in feed, thereby decreasing excessive protein degradation in the body. This leads to lower urea synthesis and excretion, ultimately reducing ammonia emissions [1]. However, other studies have reported that excessive reductions in dietary protein levels may negatively impact growth performance, fecal consistency, cooking loss, and drip loss [37]. These findings contrast with the results of the present study, possibly due to differences in feeding management strategies, such as optimizing the feed particle size and controlling environmental factors like temperature and humidity, which may have mitigated the adverse effects of a low-protein diet. In this study, no significant interaction was observed between the addition of EFA and the low-protein diet group. A possible explanation is that EFA primarily improves gut health, enhances growth performance, and increases nutrient utilization, whereas the primary function of a low-protein diet is to reduce excessive amino acid intake and nitrogen excretion [38]. Since these two mechanisms operate independently, their interaction may not have resulted in a significant synergistic effect. But some data from pigs fed a low-protein diet supplemented with an EFA (such as the ADG and nutrient digestibility) showed either improvements or enhancements compared to pigs fed a high-protein diet without EFA supplementation. This suggests that the addition of an EFA can effectively alleviate the adverse effects of a low-protein diet on growth performance.

## 5. Conclusions

This study investigates the effects of adding an EFA to the diets of fattening pigs with varying protein levels, focusing on growth performance, nutrient digestibility, gas emissions, fecal score, meat quality, and blood profile. Overall, the addition of an EFA significantly improved growth performance and nitrogen digestibility, reduced ammonia emissions, and enhanced meat quality, particularly in terms of water-holding capacity and LMA. Furthermore, the addition of an EFA notably elevated the NPY levels, which may contribute to appetite regulation, growth promotion, and improved meat quality. Moreover, EFA addition alleviated the negative impacts of low-protein diets, offering practical benefits for improving growth performance and meat quality in finishing pigs. However, further research is needed to clarify the underlying mechanisms, particularly regarding its variable effects under different physiological conditions.

## Figures and Tables

**Table 1:** Nutritional composition of the EFA.

Component	Content (Minimum)
β-glucan	≥10%
α-amylase	≥40.0 U/g
Cellulase	≥30.0 U/g
Protease	≥35.0 U/g
Glucose	≥8%
Fructose	≥6%
Sucrose	≥0.5%
*Lactobacillus plantarum*	≥1.0 × 10^6^ cfu/g
*Bacillus subtilis*	≥1.0 × 10^6^ cfu/g
*Saccharomyces cerevisiae*	≥1.0 × 10^6^ cfu/g

**Table 2:** Composition of finishing pig diets.

Item	Experimental Diet	
	Basal Diet	Basal Diet-2% CP
Ingredients (%)		
Corn	71.02	75.92
Soybean meal	13.06	7.65
DDGS	10.00	10.00
Tallow	2.65	2.74
MDCP	1.10	1.27
Limestone	0.73	0.64
Salt	0.30	0.30
Methionine (99%)	0.05	0.08
Lysine (78%)	0.50	0.69
Threonine (99%)	0.11	0.20
Tryptophan (99%)	0.05	0.08
Mineral mix ^1^	0.20	0.20
Vitamin mix ^2^	0.20	0.20
Choline (25%)	0.03	0.03
Total	100.00	100.00
Calculated value		
Cp, %	15.00	13.00
Ca, %	0.60	0.60
P, %	0.55	0.55
LYS, %	1.00	1.00
MET, %	0.30	0.30
ME, kacl/kg	3300	3300
FAT, %	6.14	6.31

^1^ DDGS: distillers dried grains with solubles; MDCP: mono dicalcium phosphate; CP: crude protein; ME: metabolizable energy; LYS: lysine; MET: methionine. Supplied per kilogram of diet: Fe, 115 mg as ferrous sulfate; Mn, 20 mg as manganese oxide; Cu, 70 mg as copper sulfate; I, 0.5 mg as potassium iodide; Zn, 60 mg as zinc oxide; and Se, 0.3 mg as sodium selenite. ^2^ Supplied per kilogram of diet: vitamin D_3_, 1700 IU; vitamin A, 13,000 IU; vitamin B_1_, 4.2 mg; vitamin E, 60 IU; vitamin K_3_, 5 mg; vitamin B_2_, 19 mg; vitamin B_6_, 6.7 mg; vitamin B_12_, 0.05 mg; niacin, 55 mg; biotin, 0.34 mg; folic acid, 2.1 mg; D-calcium pantothenate, 45 mg.

**Table 3:** The effect of the addition of EFA to diets with varying protein levels on growth performance in finishing pigs ^1^.

Protein	Low Protein		High Protein		SEM	*p*-Value		
EFA	−	+	−	+				
Items						Protein	EFA	Interaction
Body weight, kg								
Initial	55.05	55.06	55.06	55.05	1.10	0.999	0.996	0.994
Week 5	82.51 ^b^	83.30 ^ab^	82.99 ^ab^	83.73 ^a^	1.19	0.703	0.524	0.982
Week 10	114.25 ^b^	116.86 ^a^	116.11 ^ab^	117.81 ^a^	1.54	0.368	0.169	0.771
Initial-Week 5								
ADG, g	785 ^b^	807 ^ab^	798 ^ab^	819 ^a^	11	0.224	0.048	0.967
ADFI, g	2071	2107	2089	2127	22	0.373	0.098	0.973
FCR	2.640	2.612	2.620	2.597	0.02	0.3834	0.201	0.877
Week 5–10								
ADG, g	907 ^b^	959 ^a^	946 ^ab^	974 ^a^	18	0.137	0.032	0.499
ADFI, g	2640	2716	2698	2740	38	0.280	0.127	0.650
FCR	2.917	2.836	2.854	2.819	0.04	0.327	0.153	0.558
Overall								
ADG, g	846 ^b^	883 ^a^	872 ^ab^	897 ^a^	12	0.103	0.015	0.607
ADFI, g	2355	2411	2394	2433	25	0.231	0.061	0.741
FCR	2.787	2.733	2.746	2.717	0.03	0.308	0.136	0.651

^1^ Abbreviation: ADG: average daily gain; ADFI: average daily feed intake; FCR: feed conversion ratio; EFA: eco-friendly additive; SEM: standard error of means. ^a,b^ Means in the same row with different superscripts differ significantly (*p* < 0.05).

**Table 4:** The effect of the addition of EFA to diets with varying protein levels on gas emission in finishing pigs ^1^.

Protein	Low Protein		High Protein		SEM	*p*-Value		
EFA	−	+	−	+				
Items						Protein	EFA	Interaction
Week 10								
NH_3_	11.88 ^ab^	11.00 ^b^	12.75 ^a^	11.75 ^ab^	0.34	0.036	0.019	0.859
H_2_S	8.10	7.98	8.33	8.00	0.37	0.743	0.558	0.793
Methyl mercaptans	8.13	7.88	8.25	8.00	0.34	0.721	0.479	1.000
Acetic acid	7.00	6.63	7.38	6.75	0.31	0.436	0.133	0.694
CO_2_	13,600	13,450	14,100	13,775	236	0.106	0.335	0.718

^1^ Abbreviation: NH_3_: ammonia; H_2_S: hydrogen sulfide; CO_2_: carbon dioxide; EFA: eco-friendly additive; SEM: standard error of means. ^a,b^ Means in the same row with different superscripts differ significantly (*p* < 0.05).

**Table 5:** The effect of the addition of EFA to diets with varying protein levels on fecal score in finishing pigs ^1^.

Protein	Low Protein		High Protein		SEM	*p*-Value		
EFA	−	+	−	+				
Items						Protein	EFA	Interaction
Fecal score								
Week 10	3.22	3.17	3.26	3.21	0.05	0.527	0.354	1.000

^1^ Abbreviation: EFA: eco-friendly additive; SEM: standard error of means.

**Table 6:** The effect of the addition of EFA to diets with varying protein levels on meat quality in finishing pigs ^1^.

Protein	Low Protein		High Protein		SEM	*p*-Value		
EFA	−	+	−	+				
Items						Protein	EFA	Interaction
pH	5.49	5.54	5.55	5.59	0.06	0.393	0.459	1.000
WHC, %	37.56 ^b^	40.70 ^a^	39.85 ^ab^	42.41 ^a^	1.13	0.102	0.027	0.801
LMA, cm^2^	7862.25 ^b^	8039.08 ^ab^	7981.72 ^ab^	8174.15 ^a^	84.35	0.157	0.049	0.928
Meat color								
L*	56.02	55.87	56.14	56.28	0.46	0.574	0.998	0.756
a*	15.54	15.48	15.43	15.63	0.40	0.959	0.867	0.759
b*	6.53	6.75	6.69	6.86	0.28	0.644	0.512	0.938
Cooking loss, %	22.69	22.17	22.36	21.98	0.64	0.688	0.495	0.921
Drip loss, %								
d1	1.14	1.04	1.05	0.98	0.09	0.406	0.364	0.871
d3	2.14	2.04	2.05	1.95	0.08	0.301	0.228	1.000
d5	4.20	4.09	4.12	3.99	0.09	0.360	0.218	0.990
d7	5.15 ^a^	4.99 ^ab^	5.04 ^ab^	4.87 ^b^	0.06	0.084	0.022	0.904
Sensory evaluation								
Color	3.00	3.08	3.17	3.08	0.14	0.572	0.993	0.560
Marbling	2.92	3.00	3.08	3.00	0.13	0.548	0.993	0.536
Firmness	3.17	3.08	3.00	3.08	0.12	0.507	0.992	0.507
Skin color								
L*	52.14	52.97	52.75	52.85	0.40	0.266	0.552	0.376
a*	6.32	6.76	6.83	6.59	0.20	0.639	0.412	0.105
b*	13.06	12.26	12.93	12.98	0.25	0.418	0.23	0.097

^1^ Abbreviation: L*: lightness; a*: redness; b*: yellowness; EFA: eco-friendly additive; WHC: water-holding capacity; LMA: *longissimus* muscle area; SEM: standard error of means. ^a,b^ Means in the same row with different superscripts differ significantly (*p* < 0.05).

## Data Availability

The data presented in this study are available on request from the corresponding author.
